# The Use of Point-of-care Ultrasound in the Diagnosis of Pott’s Puffy Tumor: A Case Report

**DOI:** 10.5811/cpcem.2021.6.52726

**Published:** 2021-09-01

**Authors:** Josie Acuña, Daniel Shockey, Srikar Adhikari

**Affiliations:** The University of Arizona Tucson, Department of Emergency Medicine, Tucson, Arizona

**Keywords:** ultrasound, point-of-care, emergency medicine, radiology, case report

## Abstract

**Introduction:**

Pott’s puffy tumor (PPT) is a rare clinical disease characterized by forehead swelling from a subperiosteal abscess coupled with frontal bone osteomyelitis. It is often associated with severe complications and poor outcomes if left undiagnosed; thus, rapid recognition is crucial. Point-of-care ultrasound (POCUS) may provide an alternative pathway to diagnosis. It can be performed rapidly at the bedside and assist in early screening of patients, identifying those with high suspicion for PPT and prioritizing imaging and consultation.

**Case Report:**

A 59-yghb ar-old-male presented to the emergency department for evaluation of a “lump” on his forehead. He recently had a bifrontal craniotomy to de-bulk a polyp burden in an effort to manage his recurrent sinusitis. To further characterize the mass, a POCUS examination was performed by the treating emergency physician. The examination found a subcutaneous, hypoechoic fluid collection extending superficially along the frontal bone. A discontinuity in the surface of the frontal bone was visualized through which the collection appeared to extend. Given the heightened concern for PPT based on the POCUS examination findings, otolaryngology service was consulted and the patient was admitted for further imaging and treatment.

**Conclusion:**

Pott’s puffy tumor is a rare diagnosis that has the potential for life-threatening complications. Timely diagnosis is imperative. Point-of-care ultrasound can easily be used to help identify patients with suspicion for PPT in the acute care setting and influence patient management with regard to obtaining further imaging and plans for early consultation.

## INTRODUCTION

Pott’s puffy tumor (PPT) is a rare clinical disease characterized by a forehead swelling from a subperiosteal abscess associated with frontal bone osteomyelitis. The condition is commonly associated with frontal sinusitis or trauma to the forehead.^1^ Often PPT can lead to complications such as epidural abscess, subdural empyema, and brain abscess.^2^ The treatment for PPT is prompt surgical and antibiotic management; thus, rapid recognition is crucial to prevent further complications. Contrast-enhanced computed tomography is typically the initial imaging of choice for PPT in the acute care setting, followed by magnetic resonance imaging if readily available**.3** However, advanced imaging can be expensive, time prohibitive, and difficult to obtain in a resource-limited setting.

Point-of-care ultrasound (POCUS) may provide an alternative pathway to diagnosis. It can be performed rapidly at the bedside and can assist in screening these patients early on, identifying those with high suspicion for PPT and prioritizing imaging. A limited number of reports in the literature discuss the use of ultrasound as first-line imaging for PPT and primarily describe cases in pediatric patients.^4^–^6^ Even fewer reports discuss the use of POCUS performed by the provider in the acute care setting. We present this case to highlight how POCUS can be a useful modality for the rapid recognition of PPT.

## CASE REPORT

A 59-year-old-male with a history of hypertension, chronic rhinosinusitis with nasal polyps requiring several endoscopic sinus surgeries presented to the emergency department (ED) for evaluation of a “lump” on his forehead. He recently had a bifrontal craniotomy to de-bulk a polyp burden in an effort to manage his recurrent sinusitis. He first noticed the mass 10 days prior to presentation, and it had slowly grown since then. Additionally, he endorsed a constant, pressure-like headache. On arrival to the ED, vital signs demonstrated a temperature of 36.8 degrees Celsius, a heart rate of 106 beats per minute, blood pressure of 114/73 millimeters of mercury, respiratory rate of 18 breaths per minute, and oxygen saturation of 98% on room air. Physical examination revealed a 3 centimeter (cm) × 2 cm x1 cm mass located centrally on his forehead. The mass was without erythema, induration, or drainage, and was mildly tender to palpation.

Results of a complete blood cell count and complete metabolic panel were within normal limits. To further characterize the mass the treating physician performed a POCUS examination using a high-frequency, linear transducer. This exam revealed a hypoechoic, well-circumscribed area. Periosteal lifting was noted, along with an anechoic subperiosteal collection. Deep to the fluid collection, disruption of the underlying frontal bone was also appreciated. There were no sonographic signs of surrounding cellulitis or other significant findings. Given the heightened concern for PPT based on the POCUS examination, an otolaryngologist was consulted who recommend the patient be admitted for antibiotic treatment and likely surgical intervention.

## DISCUSSION

Early suspicion and diagnosis of PPT increases the chance of good recovery. Pott’s puffy tumor is a risk factor for intracranial serious complications such as subdural empyema and brain abscess**.7–8** Although rare, given the high prevalence and severity of complications associated with PPT, providers should have a heightened level of suspicious for this diagnosis. A literature review that described 92 adolescent and pediatric patients with PPT found the overall rate of intracranial complications to be 72%.^9^ Although the incidence of complications is lower among adults, literature review found that these patients were still at high risk of serious intracranial complications with a rate of 29%.^10^

**Image 1:**
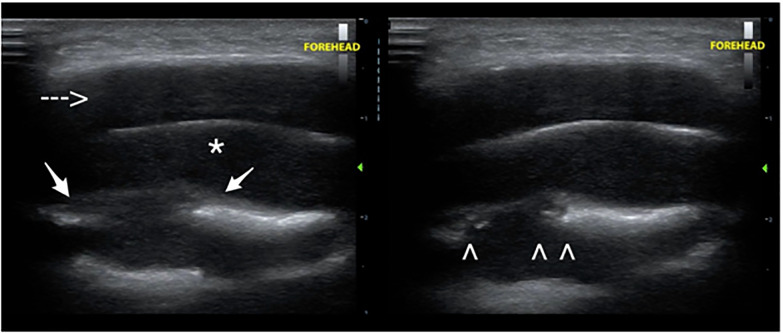
Transverse grayscale images over the area of swelling in the midline forehead demonstrating a hypoechoic superficial fluid (dashed arrow), and a subgaleal fluid collection (*) extending along the superficial aspect of the frontal bone. Periosteal lifting is noted (arrows). There is focal discontinuity in the surface of the bone (arrowheads), through which the collection appears to extend.

In instances where history and clinical signs suggest PPT, a POCUS examination can provide immediate information that can aid in the diagnosis. A linear, high-frequency transducer can be used for this examination. Both transverse and sagittal views of the frontal mass should be obtained. A PPT will appear as a hypoechoic, well-circumscribed area**.** Periosteal lifting is noted, along with an anechoic subperiosteal collection. The subperiosteal abscess will often demonstrate peripheral hypervascularity. Deep to the fluid collection, disruption of the normally smooth, linear, underlying frontal bone is appreciated, which is consistent with erosion from osteomyelitis (Image 1).

**CPC-EM Capsule**

What do we already know about this clinical entity?

*Pott’s puffy tumor (PPT) is a rare clinical entity associated with life-threatening complications. Timely diagnosis is imperative.*

What makes this presentation of disease reportable?

*This case highlights the use of point-of-care ultrasound for the rapid recognition of PPT.*

What is the major learning point?

*Point-of-care ultrasound can assist in screening patients for PPT to identify those with high suspicion for this disease and prioritize imaging.*

How might this improve emergency medicine practice?

*Point-of-care ultrasound can be used to help identify patients with suspicion for PPT in the acute care setting and influence patient management.*

Signs of cortical disruption should raise concern for intracranial extension and prompt further evaluation with other imaging modalities. Color Doppler may also be used over the area of swelling to further assess the extent of the fluid in cases where intracranial extension is unclear.

**Image 2:**
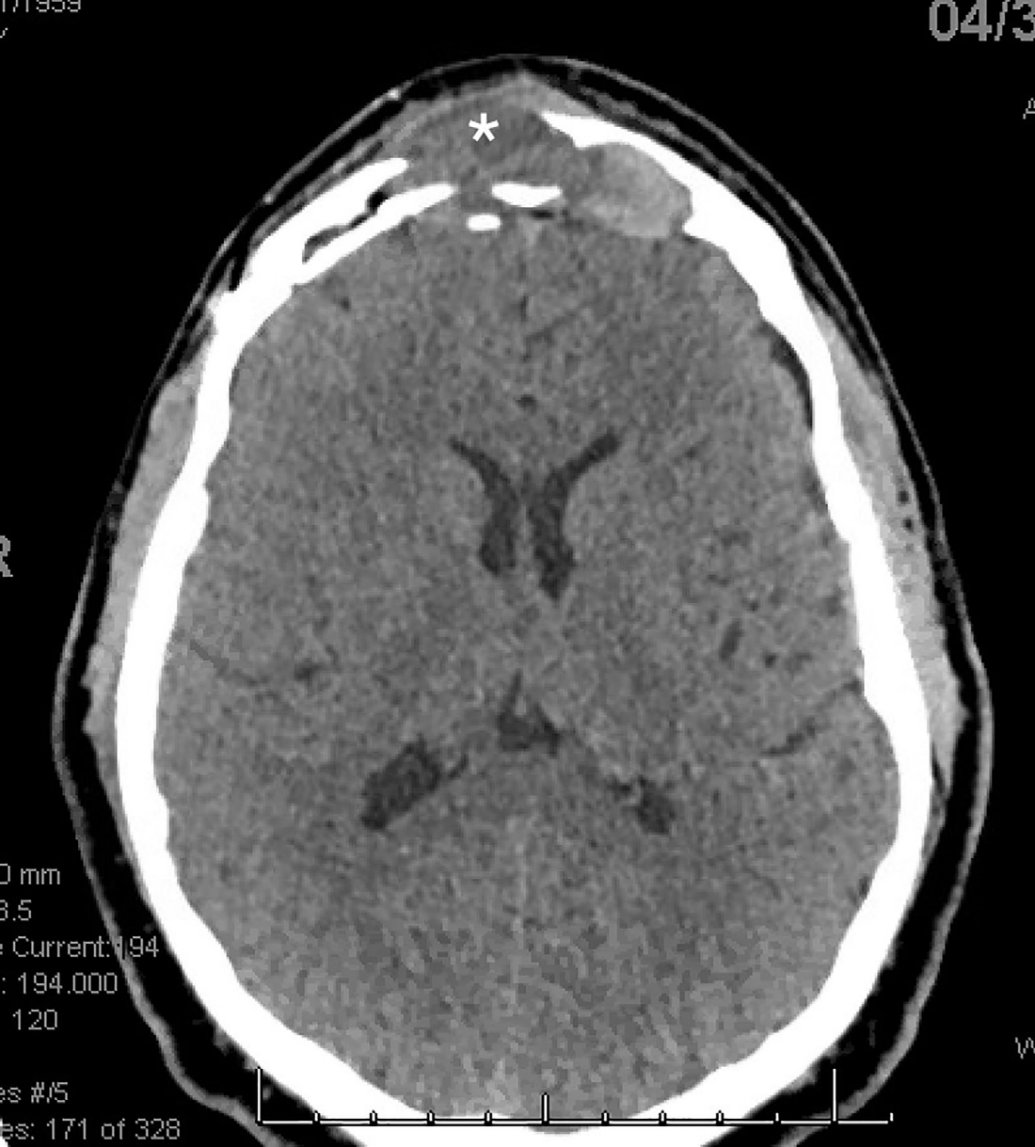
Computed tomography imaging demonstrating a soft tissue mass (*) extending through the frontal sinus and the right frontal lobe.

After evaluation of the patient in the ED by otolaryngology, a needle aspiration was performed to take a sample of fluid from the mass. Computed tomography imaging demonstrated a soft tissue mass extending throughout the frontal sinus, including extension into the right frontal lobe consistent with Pott’s puffy tumor (Image 2).

The patient was taken to the operating room emergently the next morning. This case demonstrates a unique clinical scenario of POCUS evidence of PPT. One should be aware of the inherent limitations of POCUS, such as its reliance on operator experience. However, this examination involves a technique that can be easily learned by most emergency physicians. Using POCUS as an initial imaging modality in the acute care setting can allow providers to risk-stratify patients, determine the need for other imaging modalities, and obtain consultation more quickly.

## CONCLUSION

Pott’s puffy tumor is a rare diagnosis that has the potential for life-threatening complications. Timely diagnosis is imperative. Point-of-care ultrasound can easily be used to help identify patients with suspicion for PTT in the acute care setting and influence patient management with regard to obtaining further imaging and plans for early consultation.
